# Effects of different signaling pathways on odontogenic differentiation of dental pulp stem cells: a review

**DOI:** 10.3389/fphys.2023.1272764

**Published:** 2023-10-19

**Authors:** Lisha Zhou, Shuya Zhao, Xianghui Xing

**Affiliations:** Department of Pediatric Dentistry, Nanjing Stomatological Hospital, Affiliated Hospital of Medical School, Nanjing University, Nanjing, China

**Keywords:** dental pulp stem cell, odontogenic differentiation, signaling pathway, pulp–dentin complex regeneration, tissue engineering, review

## Abstract

Dental pulp stem cells (DPSCs) are a type of mesenchymal stem cells that can differentiate into odontoblast-like cells and protect the pulp. The differentiation of DPSCs can be influenced by biomaterials or growth factors that activate different signaling pathways *in vitro* or *in vivo*. In this review, we summarized six major pathways involved in the odontogenic differentiation of DPSCs, Wnt signaling pathways, Smad signaling pathways, MAPK signaling pathways, NF-kB signaling pathways, PI3K/AKT/mTOR signaling pathways, and Notch signaling pathways. Various factors can influence the odontogenic differentiation of DPSCs through one or more signaling pathways. By understanding the interactions between these signaling pathways, we can expand our knowledge of the mechanisms underlying the regeneration of the pulp–dentin complex.

## 1 Introduction

Dental pulp stem cells (DPSCs) are a type of mesenchymal stem cells (MSCs) that express mesenchymal markers, including CD29, CD44, CD59, CD90, CD105, CD146, and CD166 (Liu et al., 2015). Since their discovery and characterization by Gronthos et al. (2000), DPSCs have attracted great interest in the field of regenerative medicine. This is due to their ability to self-renew, their high proliferation rate, their immunomodulatory properties, and their versatile potential to differentiate into different cell types. Furthermore, DPSCs can be easily obtained from extracted third molars or primary teeth. Dental tissues contain various types of MSC sources, namely, pulp, follicle, and papilla, which can differentiate into the desirable lineages when induced under appropriate conditions (Gronthos et al., 2000). DPSCs are used in the treatment of Alzheimer’s disease, diabetes, and immunological diseases and have shown the ability to differentiate into angioblasts and neural cells (Marchionni et al., 2009; Jahanbin et al., 2016; Nuti et al., 2016), smooth muscle cells (Song et al., 2016; Jiang et al., 2019; Ha et al., 2021), chondroblasts (Ba et al., 2017), or hepatocytes (Lei et al., 2021; Li et al., 2021). Most importantly, DPSCs have the unique capability to replace damaged odontoblasts and generate reparative dentin. When odontoblasts are stimulated by dental caries or trauma, DPSCs migrate to the lesion site and differentiate into odontoblast-like cells that contribute to the formation of dentin and pulp-like structures (Arana-Chavez and Massa, 2004; Janebodin et al., 2011; Ma et al., 2012). Therefore, DPSCs play a crucial role in the regeneration of the pulp–dentin complex (Xuan et al., 2018). Pulp disease is a common cause of tooth loss in young individuals (Hull et al., 1997). Although root canal treatment has been proven effective in the treatment of pulp diseases (Oviir, 2005), it does have some limitations, such as tooth discoloration or thick dentinal walls (Jeeruphan et al., 2012). To solve this problem, it is important to focus on regenerating the pulp–dentin complex. However, the odontogenic differentiation of DPSCs is extremely complex and involves multiple signaling pathways. Currently, a large number of studies have shown that activation of associated signaling pathways can promote the odontogenic differentiation of DPSCs. However, there is little comprehensive literature summarizing the signaling pathways related to the odontogenic differentiation of DPSCs (Liang et al., 2021). To provide insight into the better utilization of DPSCs as “seed cells” for pulp–dentin complex regeneration, this article aims to summarize the known signaling pathways associated with the odontogenic differentiation of DPSCs.

## 2 Results

### 2.1 Wnt signaling pathways

**FIGURE 1:**
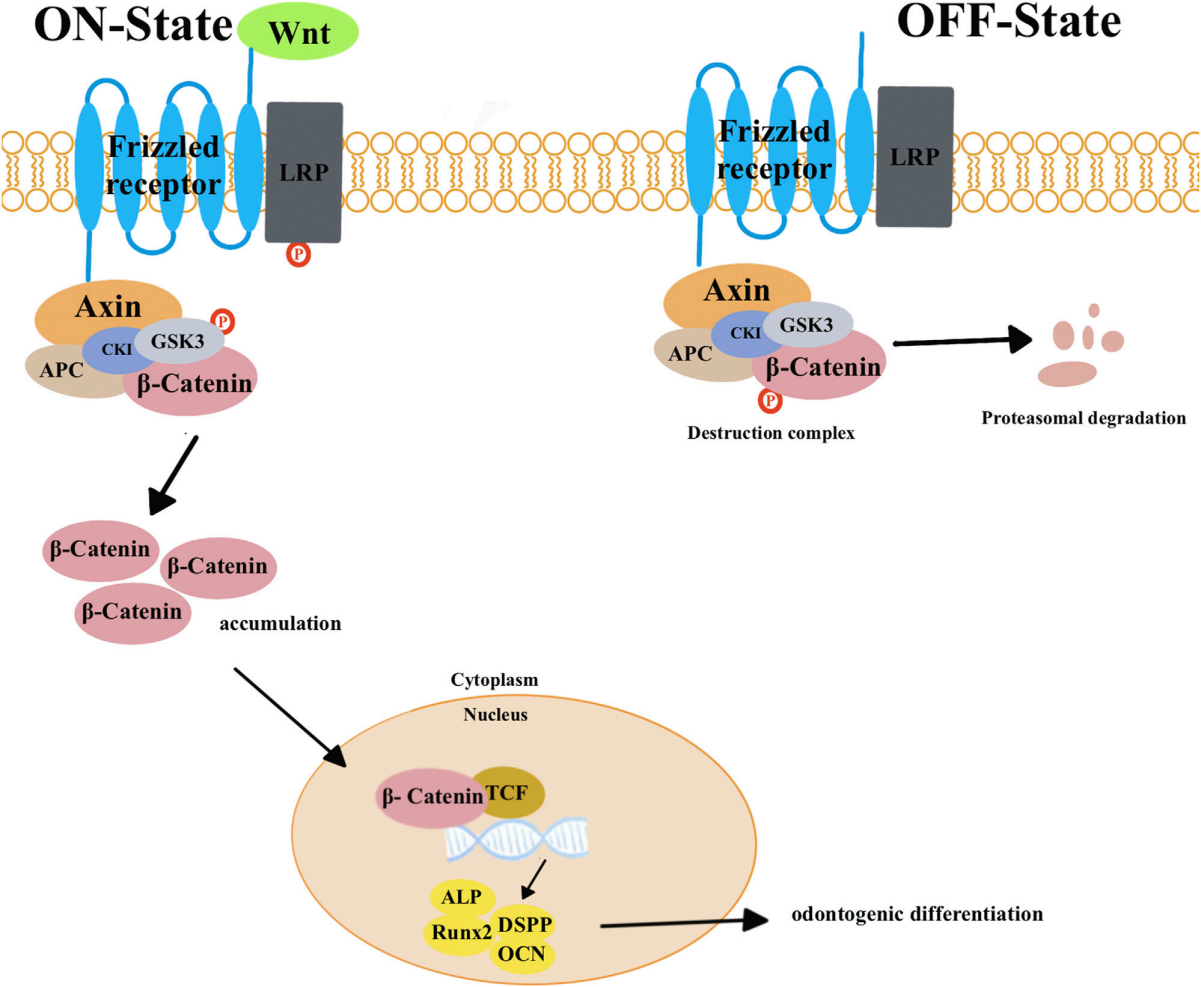
In canonical pathways, the presence of *Wnt* triggers a series of events. This begins with the binding of the frizzled receptor and the low-density lipoprotein receptor-related proteins 5 and 6 (*LRP5/6*). This binding transmits signals that lead to the degradation of the complex consisting of adenomatous polyposis coli (*APC*), glycogen synthase kinase-3β (*GSK-3β*), *Axin*, and casein kinase 1 (*Ck1*). This causes *β-catenin* to be released from this complex, resulting in an increase in its level. The increased *β-catenin* then moves into the nucleus, displaces co-repressors from transcription factors (*TCFs*), and activates target genes and proteins involved in odontogenic differentiation, such as *Runx2*, *ALP*, *DSPP*, and *OCN*. When *Wnts* are absent, *β-catenin* remains associated with a protein complex that includes *APC*, *GSK3β*, *Axin*, and *Ck1*. Within this complex, *GSK-3β* phosphorylates *β-catenin* to degrade it. This degradation prevents the occurrence of signal transduction in the Wnt signaling pathway.

Wnt signaling pathways play a critical role in promoting tissue repair and regeneration. These pathways can be divided into two types: canonical and non-canonical pathways (Rao and Kuhl, 2010). In canonical pathways (Figure 1), the absence of Wnts leads to the formation of a protein complex consisting of adenomatous polyposis coli (*APC*), glycogen synthase kinase-3*β* (*GSK-3β*), *Axin*, and casein kinase 1 (*Ck1*). This complex then phosphorylates *β-catenin* and leads to its degradation. This inhibits signal transduction in the Wnt signaling pathway. When *Wnts* are present, they interact with the frizzled receptor and the low-density lipoprotein receptor-related proteins 5 and 6 (*LRP5/6*). This interaction leads to the destruction of the protein complex. This allows cytoplasmic *β-catenin* to accumulate and translocate into the nucleus. Once in the nucleus, *β-catenin* activates target genes and proteins that are involved in odontogenic differentiation, such as *Runx2*, *ALP*, *DSPP*, and *OCN*. In addition to the canonical pathways, there are also non-canonical pathways that are mediated by *Wnt5a*. The protein *β-catenin* is not involved in these pathways. Instead, non-canonical pathways are divided into two subtypes: the planar cell polarity (PCP) pathways and the Wnt/Ca^2+^ pathways. These non-canonical pathways play a role in tissue repair and regeneration as well (Monroe et al., 2012; He et al., 2014).

Numerous studies have been conducted to investigate how Wnt signaling pathways affect the odontogenic differentiation of DPSCs. However, differences in results are noted. Some researchers have found that *β-catenin* can activate Runt-related transcription factor 2, (*Runx2*) and the accumulation of *β-catenin* can enhance the odontogenic differentiation of DPSCs (Kim et al., 2013; Han et al., 2014; Yoshida et al., 2016; Rahman et al., 2018). In another study, Xin et al. transfected wild-type and mutant special AT-rich sequence-binding protein 2 (*SATB2*) into DPSCs to induce odontogenic differentiation. The results showed that DPSCs transfected with wild-type SATB2 increased the expression of *Runx2*, osteopontin (*OPN*), and alkaline phosphatase (*ALP*) and also improved calcium nodule formation ability compared to those transfected with mutant *SATB2*. Dickkopf-1 (*DKK1*) is considered to be an inhibitor of the Wnt/β-catenin signaling pathways. Compared with wild-type ones, DPSCs that were transfected with mutant SATB2 increased the expression of *DKK1* and decreased the expression of active *β-catenin*. Treated with XAV939 (inhibitor of Wnt signaling pathways), the expression of *ALP* and *Runx2* was decreased both in wild-type and mutant SATB2 transfection groups. This indicates that wild-type SATB2 enhanced the odontogenesis of DPSCs through the Wnt/β-catenin signaling pathways (Xin et al., 2021). Chen et al. revealed that overexpression of differentiation antagonizing non-protein coding RNA (DANCR) suppressed the formation of mineralized nodules and the expression of dentin sialophosphoprotein (*DSPP*) and dentin matrix protein 1 (*DMP-1*) in DPSCs after 14 days of odontogenic induction. Meanwhile, the expression of *β-catenin* and the phosphorylation level of *GSK-3β* were significantly decreased. Therefore, DANCR inhibits the differentiation of DPSCs into odontoblast-like cells by blocking the canonical Wnt/*β-catenin* signaling pathways (Chen et al., 2016). Han et al. found that during the odontoblastic differentiation of DPSCs, the expression of *β-catenin* was upregulated. Knocking down *β-catenin* accumulation in DPSCs using lentivirus resulted in a significant reduction in matrix mineralization and calcium nodule formation and the expression of *DSPP*, *DMP-*1, and *ALP*. Conversely, stimulating *β-catenin* accumulation in DPSCs using LiCl led to a significant enhancement in calcium nodule formation and matrix mineralization and the expression of *DSPP*, *DMP-1*, *ALP*, and osteocalcin (*OCN*) (Han et al., 2014).

These findings suggested that when a special factor promotes the odontogenic differentiation of DPSCs, it can enhance the activity of proteins related to the Wnt signaling pathways. Therefore, these results supported the idea that Wnt signaling pathways can promote the differentiation of DPSCs (Yamashiro et al., 2007; Yokose and Naka, 2010; Lim et al., 2014; Rahman et al., 2018; Lu et al., 2019). Activation of the Wnt/β-catenin signaling pathways in DPSC cultures leads to a significant increase in the expression of important core pluripotency factors, such as *SOX2*, *SSEA1*, *LIN28*, *NANOG*, and *REX1*. As a result, the ability of DPSCs to proliferate, self-renew, and generate mature cell types becomes more efficient (Uribe-Etxebarria et al., 2017). The analysis of the metabolic and epigenetic changes revealed that activation of Wnt signaling pathways upregulated cellular consumption of glucose and glutamate/glutamine by DPSCs along with a higher mitochondrial activity (Uribe-Etxebarria et al., 2019). In addition, this transient “hyper-energetic” state appears to be crucial for maintaining the stemness of the cells.

On the contrary, some studies have shown that the Wnt/β-catenin signaling pathway could inhibit the odontogenic differentiation of DPSCs. They have found that a specific protein associated with the Wnt signaling pathway can suppress the odontogenic differentiation of DPSCs (Amri et al., 2016). ScheHer et al. demonstrated that *Wnt-1* and *β-catenin* inhibited the odontogenic differentiation of DPSCs. They observed that DPSCs overexpressing *Wnt1* and *β-catenin* showed reduced *ALP* activity after undergoing odontoblastic differentiation compared to control cells (Scheller et al., 2008). Zhang et al. found that overexpression of *Wnt10A* promotes the proliferation of DPSCs but hinders their odontoblastic differentiation. After 6-day odontogenic differentiation, *Wnt10A*-overexpressing DPSCs downregulated the expression of *DSPP*, *DMP-1*, *ALP*, and collagen-type I-alpha1 (*COL1A1*) (Zhang et al., 2014).

It seems that different molecules that control Wnt signaling pathways can have varying effects. ScheHer et al. enhanced the expression of *β-catenin* by retrovirus infection, while others activated *β-catenin* by exogenous application. This suggests that transient activation of *β-catenin* can induce odontogenic differentiation of DPSCs. Differences in the level of *Wnt* activity, experimental conditions, timing, or duration of activation could contribute to these variations (Vijaykumar et al., 2021). Vijaykumar et al. also discovered that activating Wnt/β-catenin signaling could enhance the number of BSP-GFP^+^ osteoblasts in dental pulp cultures, which aligns with other research findings (Kim et al., 2013; Teufel and Hartmann, 2019). Despite the variations in results, it still indicates that the Wnt/β-catenin signaling pathways are involved in regulating the differentiation of DPSCs, and the molecular mechanisms still need to be further studied in *in vivo* testing.

### 2.2 BMP/Smad signaling pathways

**FIGURE 2:**
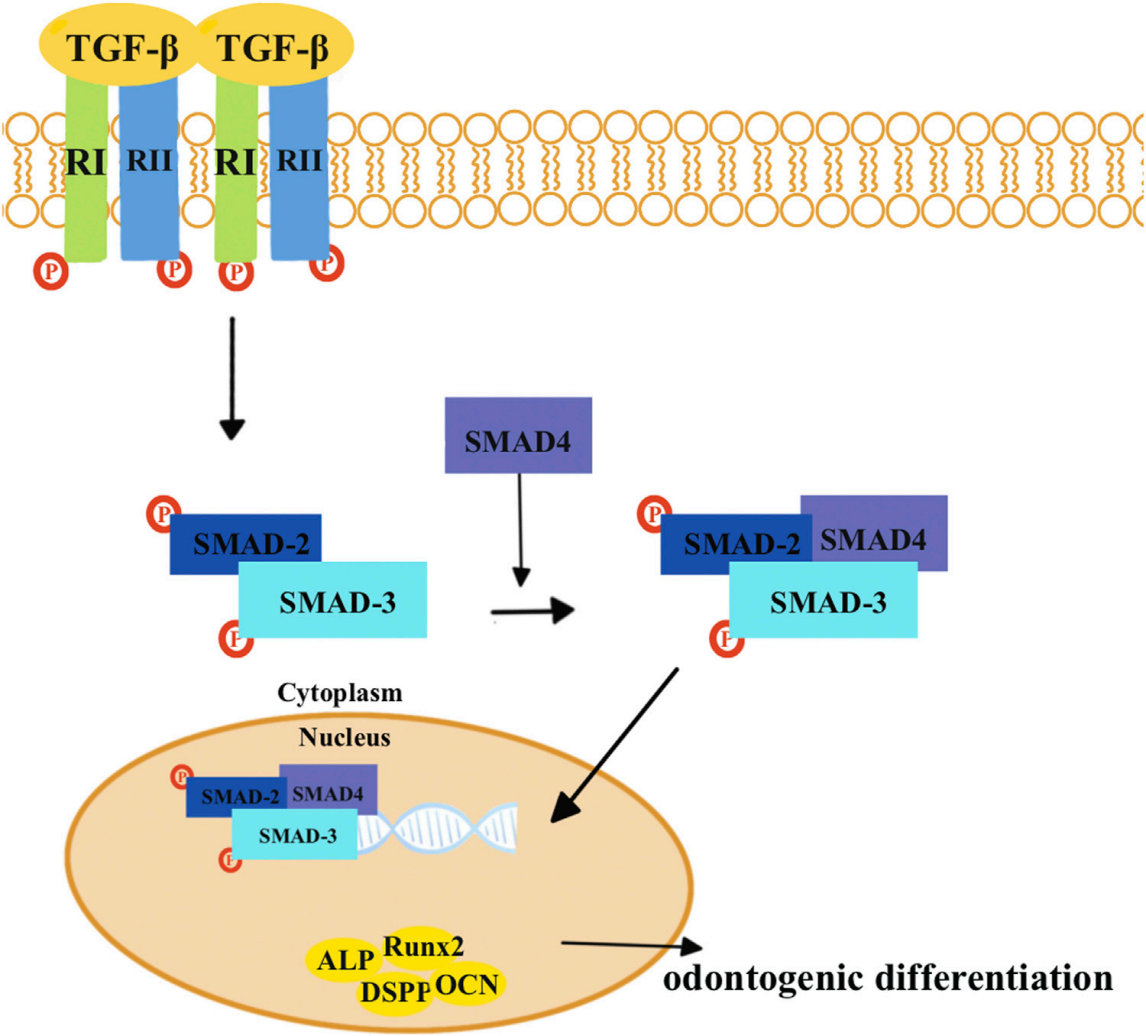
*TGF-β* combines with the *TGF-β* type II receptors (RIIs), which leads to the recruitment of *TGF-β* type I receptors (RIs). Then, two RIIs and two RIs form a heterotetrameric complex. The RI then recruits and phosphorylates *SMAD2* and *SMAD3*, which can form heteromeric complexes with *SMAD4*. Eventually, these complexes translocate into the nucleus where they activate specific genes and proteins that are involved in odontogenic differentiation, including *Runx2*, *ALP*, *DSPP*, and *OCN*.

**FIGURE 3:**
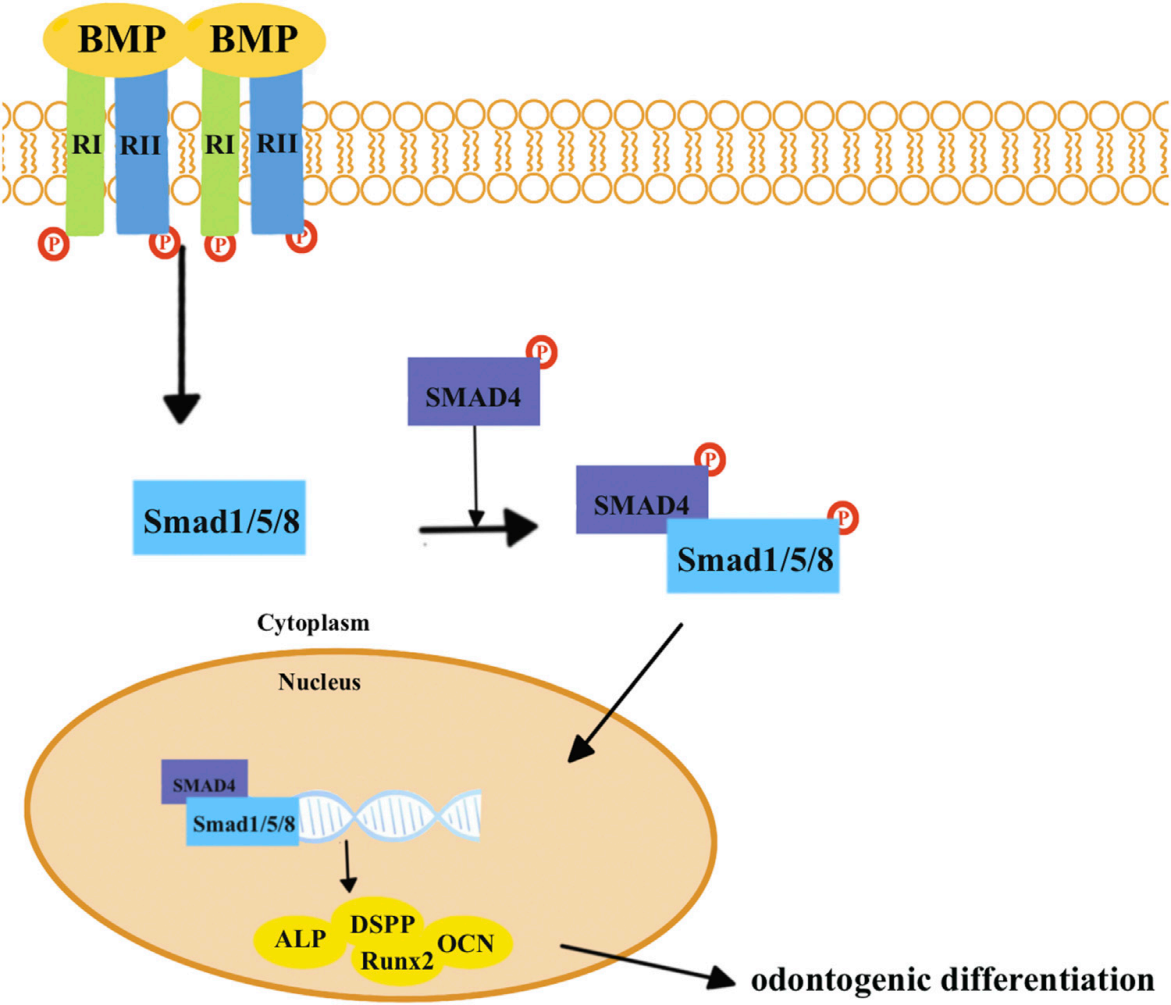
*BMP* combines with the BMP type I receptors (RIs) and TGF-β type II receptors (RIIs). *Smad1*, *Smad5*, and *Smad8* are activated by *BMP* receptors, which can form heteromeric complexes with *SMAD4*. Eventually, these complexes translocate into the nucleus to activate target genes and proteins related to odontogenic differentiation, including *Runx2*, *ALP*, *DSPP*, and *OCN*.

The transforming growth factor-β (*TGF-β*) superfamily transduces intracellular signals via *Smad* proteins (Figure 2). The bone morphogenetic protein (*BMP*), which belongs to the *TGF-β* superfamily, is most closely related to the *Smad* proteins (Figure 3) (Wu et al., 2016). *BMP* activates the *Smad* proteins, allowing signals to be transported to the nucleus for regulation of gene expression (Wu et al., 2016). *BMP* plays an important role in the process of tooth development, and inhibiting its function can lead to tooth development disorders (Fraser et al., 2004; Salazar et al., 2016; Lowery and Rosen, 2018). Previous studies have shown that BMP/Smad signaling pathways regulate *DSPP* both *in vitro* and *in vivo* (Chen et al., 2008; Chen et al., 2009; Cho et al., 2010). *BMP2* can induce DPSCs to differentiate into odontoblasts, thereby achieving dentin regeneration (Woo et al., 2016; Hu et al., 2019; Ho et al., 2021).

Zhu et al. discovered that *BMP-7* induced odontogenic differentiation of DPSCs. After 7-day and 14-day odontogenic differentiation, DPSCs that were treated with BMP-7 showed an increase in the expression of *DSPP*, *DMP-1*, *ALP*, and *OCN*. Meanwhile, Western blot results revealed that *BMP-7* induction increased the expression of *Smad5* and *p-Smad5* proteins (Zhu et al., 2018). Qin et al. concluded that *BMP2*-induced odontoblastic differentiation of DPSCs was mediated by the activated Smad1/5 signaling pathways. DPSCs exposed to BMP-2 showed an enhancement in the phosphorylation of Smad1/5 and an increase in the expression of *DSPP* and *DMP-1*. However, these increased expressions were suppressed by noggin, which was an inhibitor of BMP/Smad signaling pathways. The results also revealed that the phosphorylation and nuclear translocation of *Smad1/5* were partially inhibited by noggin (Qin et al., 2012b).

These studies have shown that *BMP* can stimulate the differentiation of DPSCs into odontoblasts. The protein associated with the Smad signaling pathways is also enhanced, which is consistent with the finding of other research studies (Nakashima and Reddi, 2003; Lin et al., 2007; Moioli et al., 2007; Yang et al., 2012). When the BMP/Smad signaling pathways are inhibited, it can negatively affect tooth development (Yamamoto et al., 2004). It has been reported that rhBMP-2 is involved in odontoblast differentiation *in vitro* (Saito et al., 2004; Casagrande et al., 2010). Therefore, it is clear that the BMP/Smad signaling pathways play a crucial role in the regeneration of the pulp–dentin complex.

### 2.3 Mitogen-activated protein kinase signaling pathways

The MAPK signaling pathways present in the cytoplasm play an important role in cell proliferation, differentiation, and apoptosis (English and Cobb, 2002). A typical *MAPK* cascade is mainly composed of three kinases, a *MAPK* (*MPK*), a *MAPK* kinase (*MAPKK or MEK*), and a *MAPK* kinase kinase (*MAPKKK or MEKK*). When a stimulus is detected, *MAPKKK*s phosphorylate and activate downstream *MAPKK*s, which then phosphorylate and activate the *MAPKs*. In turn, the activated *MAPK*s can phosphorylate numerous downstream substrates and activate cellular responses (Chen et al., 2021; Zhang and Zhang, 2022). The *MAPK* family is composed of three main subfamilies: extracellular signal-regulated kinase (*ERK*), *P38MAPK*, and c-Jun N-terminal kinase (*JNKMAPK*) (Pearson et al., 2001).

Ngo et al. concluded that leptin can stimulate the differentiation of DPSCs into odontoblasts by activating the MAPK signaling pathways. When DPSCs were treated with leptin, there was an increase in alkaline phosphatase (ALP) expression and mineralization, as well as the phosphorylation levels of *ERK*, *P38MAPK*, and *JNK*. However, leptin-induced *DSPP* protein expression levels and mineralization in DPSCs were blocked by *ERK*, *JNK*, or *P38* inhibitors (Ngo et al., 2018). Cui et al. discovered that epiregulin enhanced the odontoblastic differentiation of DPSCs by MAPK signaling pathways. Treatment with recombinant human EREG led to an increase in the expression levels of odontogenic differentiation markers in DPSCs, as well as upregulation of phosphorylated *P38MAPK* and *ERK*. However, when EREG was knocked down using lentiviral EREG short hairpin RNAs or when P38MAPK or ERK was inhibited with specific inhibitors, the expression of *DSPP*, *OCN*, and *Runx2* in DPSCs was reduced (Cui et al., 2019).

According to the aforementioned studies, when certain factors promote the odontogenic differentiation of DPSCs, the related protein of MAPK signaling pathways could be enhanced. MAPK signaling pathways are one of the crucial mechanisms that regulate cell proliferation and odontogenic differentiation (Liu et al., 2014; He et al., 2015; Sun et al., 2015; Lv et al., 2016; Rodriguez-Carballo et al., 2016; Wu et al., 2019; Cui et al., 2019). It has been reported that BMP-2, biodentine, and MTA can promote the odontogenic differentiation of DPSCs through activating MAPK signaling pathways (Qin et al., 2012a; Zhao et al., 2012; Luo et al., 2014; Sanz et al., 2021). The ERK signaling pathway, which is one of the characteristic MAPK signaling pathways, is involved in the regulation of cytodifferentiation. The P38MAPK signaling pathways are responsible for regulating cytokine expression and are activated by inflammatory cytokine signals (Su et al., 2019). JNKMAPK signaling pathways are stress-activated kinases associated with anti-proliferative and apoptotic functions (Johnson and Lapadat, 2002). However, further research is necessary to figure out the detailed mechanisms of the ERK P38 and JNK signaling pathways as well as their relationships.

### 2.4 Nuclear factor-kappa B signaling pathways

**FIGURE 4:**
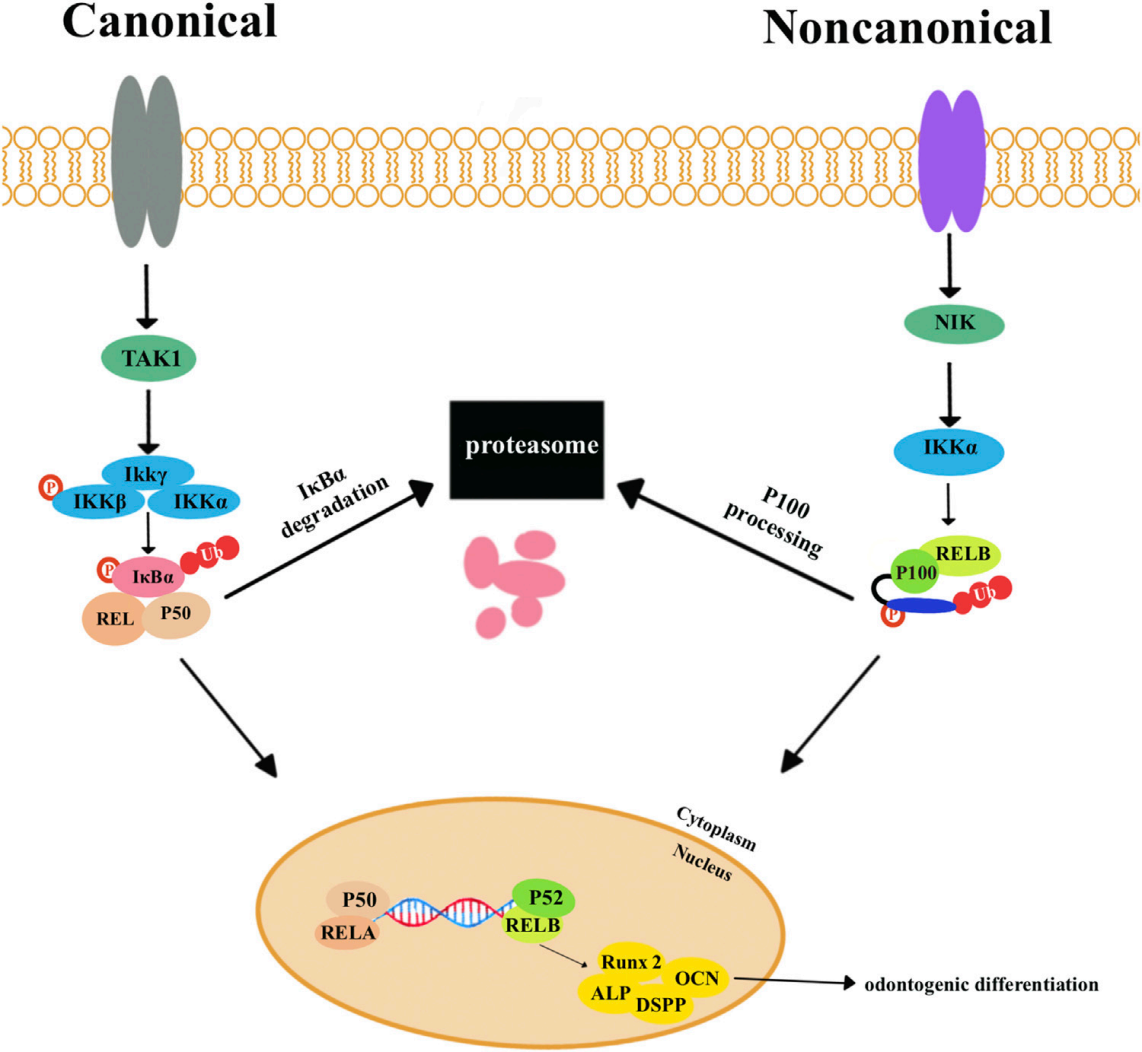
The canonical nuclear factor-κB (*NF-κB*) signaling pathways are activated by signals from various immune receptors. These signals activate the kinase TGFβ-activated kinase 1 (*TAK1*). *TAK1* phosphorylates IκB kinase-β (*IKKβ*) to activate the trimeric IκB kinase (*IKK*) complexes, which are composed of two catalytic subunits (*IKKα* and *IKKβ*) and one regulatory subunit (*IKKγ*). *IκB* family members, particularly *IκBα*, are phosphorylated by *IKK* complexes and then undergo ubiquitylation and proteasomal degradation. Eventually, various *NF-κB* complexes, predominantly the *p50*/*RELA* dimers, translocate to the nucleus. Non-canonical NF-κB signaling pathways depend on phosphorylation-induced *p100* processing, which is activated by signals from a subset of tumor necrosis factor receptor (*TNFR*) members. *TNFR* activates kinase NFκB-inducing kinase (*NIK*). *NIK* phosphorylates and triggers *IKKα*, which can phosphorylate the carboxy-terminal serine residues of *p100*, resulting in the degradation of the C-terminal IκB-like structure of *p100*. P52 and *RELB* translocate to the nucleus to activate target genes and proteins related to odontogenic differentiation, including *Runx2*, *ALP*, *DSPP*, and *OCN*.

The NF-κB signaling pathways are involved in various biological processes, such as cell proliferation and apoptosis, inflammation, and immune response. The *NF-κB* proteins usually exist as inactive cytoplasmic complexes associated with inhibitors of the κB (*IκB*) family. NF-κB signaling pathways are divided into canonical and non-canonical signaling pathways (Figure 4). In canonical pathways, signals from numerous immune receptors can activate the TGFβ-activated kinase 1 (*TAK1*). *TAK1* then phosphorylates IκB kinase-β (*IKK*β), which in turn activates a complex called IκB kinase (*IKK*). The IKK complex is made up of two catalytic subunits (*IKKα* and *IKKβ*) and one regulatory subunit (*IKKγ*). Then, *IκB* family members, particularly *IκBα*, are phosphorylated by the *IKK* complex and eventually undergo ubiquitylation and proteasomal degradation. Consequently, various *NF-κB* complexes, predominantly the *p50/RelA* dimers, translocate to the nucleus.

The non-canonical pathway responds primarily to the members of the tumor necrosis factor receptor (*TNFR*), which can activate the kinase NF-κB-inducing kinase (*NIK*). *IKKα* is phosphorylated and activated by *NIK*, which then phosphorylates and degenerates *p100* and generates *p52*. P52 and *RELB* translocate to the nucleus to activate target genes and proteins related to odontogenic differentiation, including *Runx2*, *ALP*, *DSPP*, and *OCN* (Sun, 2011; Sun, 2017).

Wang et al. discovered that estrogen deficiency decreased the odonto/osteogenic capacity of DPSCs via the NF-κB signaling pathways (Wang et al., 2013). They established an estrogen-deficient rat model by bilateral ovariectomy (OVX). The results revealed that compared to DPSCs from the sham-operated group (sham DPSCs), the odonto/osteogenic differentiation capacity of OVX-DPSCs was significantly decreased both *in vitro* and *in vivo*. Meanwhile, suppression of NF-κB signaling pathways could enhance the reduced odonto/osteogenic potential in ovariectomized mice. Li et al. (2023) confirmed that baicalin can promote odonto/osteogenic differentiation of dental pulp inflammatory stem cells by inhibiting NF-κB signaling pathways.

According to the aforementioned studies, when a certain factor promotes the odontogenic differentiation of DPSCs, the related protein of NF-κB signaling pathways could be enhanced (Hozhabri et al., 2015; Pei et al., 2016; Wu et al., 2019). It has also been found that activation of NF-κB can inhibit the odontoblast differentiation in an inflammatory microenvironment (Pei et al., 2016). Therefore, NF-κB signaling pathways do play an important role in the odontogenic differentiation of DPSCs.

### 2.5 Phosphoinositide-3-kinase/protein kinase B/mammalian target of rapamycin signaling pathways

**FIGURE 5:**
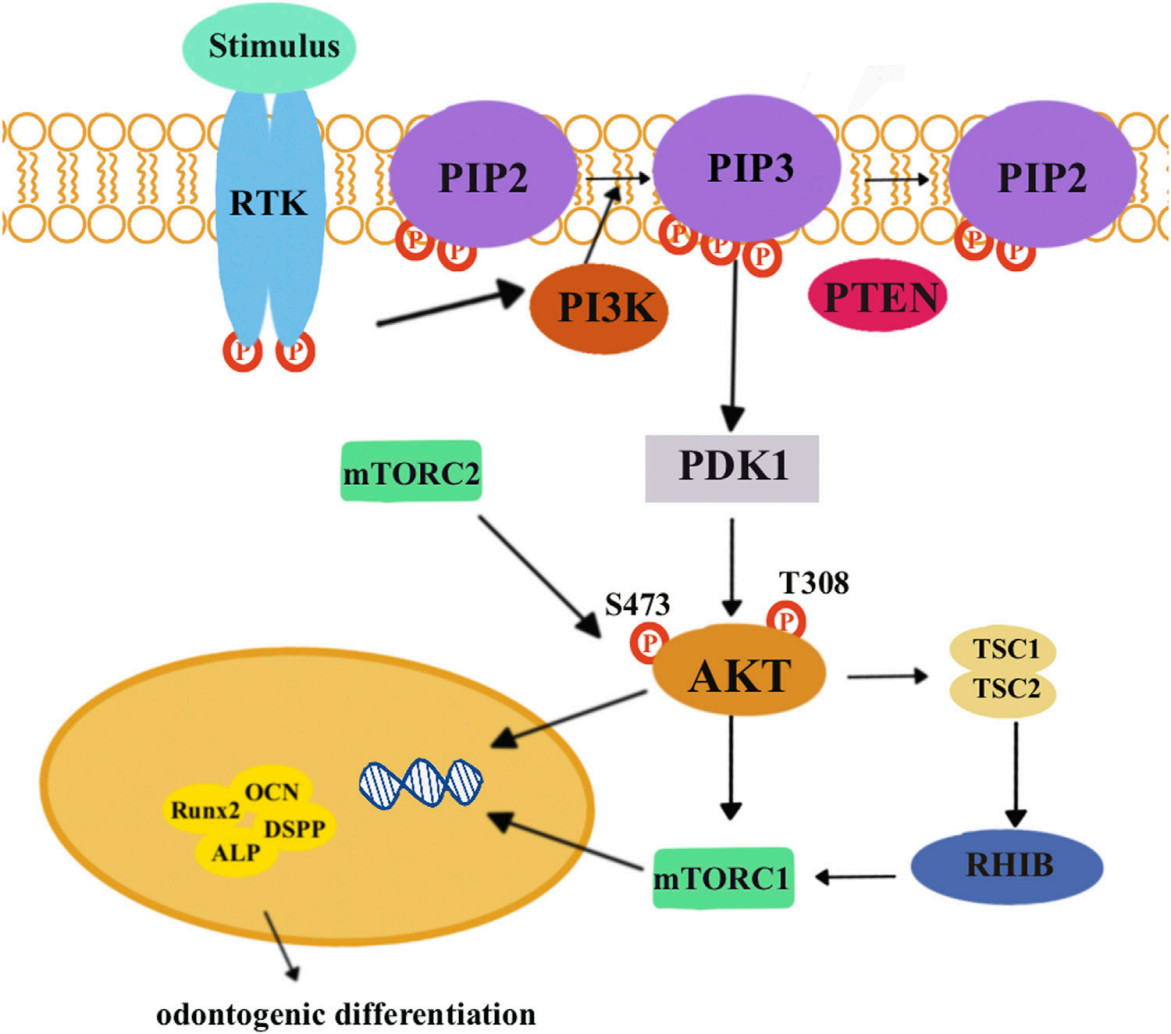
When growth factor stimulus combines with receptor tyrosine kinase (*RTK*), it activates a protein called *PI3K*. This activated PI3K can then phosphorylate phosphatidylinositol (4,5)-bisphosphate (*PIP2*) to phosphatidylinositol (3,4,5)-triphosphate (*PIP3*). *PIP3* can be further dephosphorylated by tensin homolog (*PTEN*) and activate PI3K-dependent kinase 1 (*PDK1*). *PDK1*, along with another protein called *mTORC2*, can phosphorylate specific amino acid residues, T308 and S473, respectively, to activate *AKT*. Eventually, activated *AKT* can phosphorylate target proteins to promote cell growth and survival and metabolism. Activated *AKT* inhibits inactive tuberous sclerosis complexes 1 and 2 (*TSCs* 1 and 2), which are unable to bind the RAS homolog enriched in the brain (*RHEB)*. Then, *mTORC1* is activated and exerts its effects on downstream target genes and proteins related to odontogenic differentiation, including *Runx2*, *ALP*, *DSPP*, and *OCN*.

The PI3K/AKT/mTOR signaling pathways (Figure 5) are essential for cellular processes, including cell growth, survival, and metabolism. These pathways involve key components including *PI3K*s, *AKT*, and *mTOR*. When growth factor stimulus combines with a receptor tyrosine kinase (*RTK*), activated *PI3K* can convert phosphatidylinositol 4,5-bisphosphate (*PIP2*) into phosphatidylinositol 3,4,5-triphosphate (*PIP3*). This conversion can be reversed by the phosphatase and tensin homolog (*PTEN*). *PIP3* then activates PI3K-dependent kinase 1 (*PDK1*). Subsequently, *PDK1* and *mTORC2* will, respectively, phosphorylate amino acid residues *T308* and *S473*, leading to the activation of *AKT* to phosphorylate target proteins (Keppler-Noreuil et al., 2016; Yu and Cui, 2016).

Lin et al. discovered that low concentrations of graphene oxide quantum dots (GOQDs) could enhance the odontoblastic differentiation of DPSCs. After 14-day odontoblastic differentiation, the expression level of phosphorylated AMPK increased, while the expression level of phosphorylated mTOR decreased. This indicated that GOQDs would activate the AMPK signaling pathways but inhibit the mTOR signaling pathways in DPSCs (Lin et al., 2023). Xu et al. (2021) found that NaF promoted osteo/odontogenic differentiation of DPSCs by suppressing the PI3K/AKT/mTOR signaling pathways. KEGG analysis and the results of PCR and Western blotting revealed that there was a differential expression in the PI3K/AKT/mTOR signaling pathways between NaF-treated DPSCs and the control group. The p-AKT/AKT ratio was lower in NaF-treated DPSCs. After inhibiting the PI3K/AKT/mTOR signaling pathways, NaF-treated DPSCs upregulated the expression of DSPP and DMP-1 and mineralization.

The aforementioned studies indicated that when a certain factor promotes the odontogenic differentiation of DPSCs, the related proteins of the PI3K/AKT/mTOR signaling pathways decreased. They concluded that the PI3K/AKT/mTOR signaling pathways negatively regulated the odontogenic differentiation of DPSCs. Osteogenic/dentinogenic differentiation of stem cells from apical papilla (SCAP) could be promoted by inhibiting PI3K/AKT/mTOR signaling pathways (Bhaskar and Hay, 2007). Consistently, odontogenic differentiation of DPSCs could be blocked by PI3K/AKT/mTOR signaling pathways (Kajiura et al., 2021; Park et al., 2022). However, PI3K/AKT/mTOR signaling pathways could promote the osteogenic differentiation of periodontal ligament stem cells (Huang et al., 2021). Considering this difference, we need further studies to figure out the mechanisms.

### 2.6 Notch signaling pathways

**FIGURE 6:**
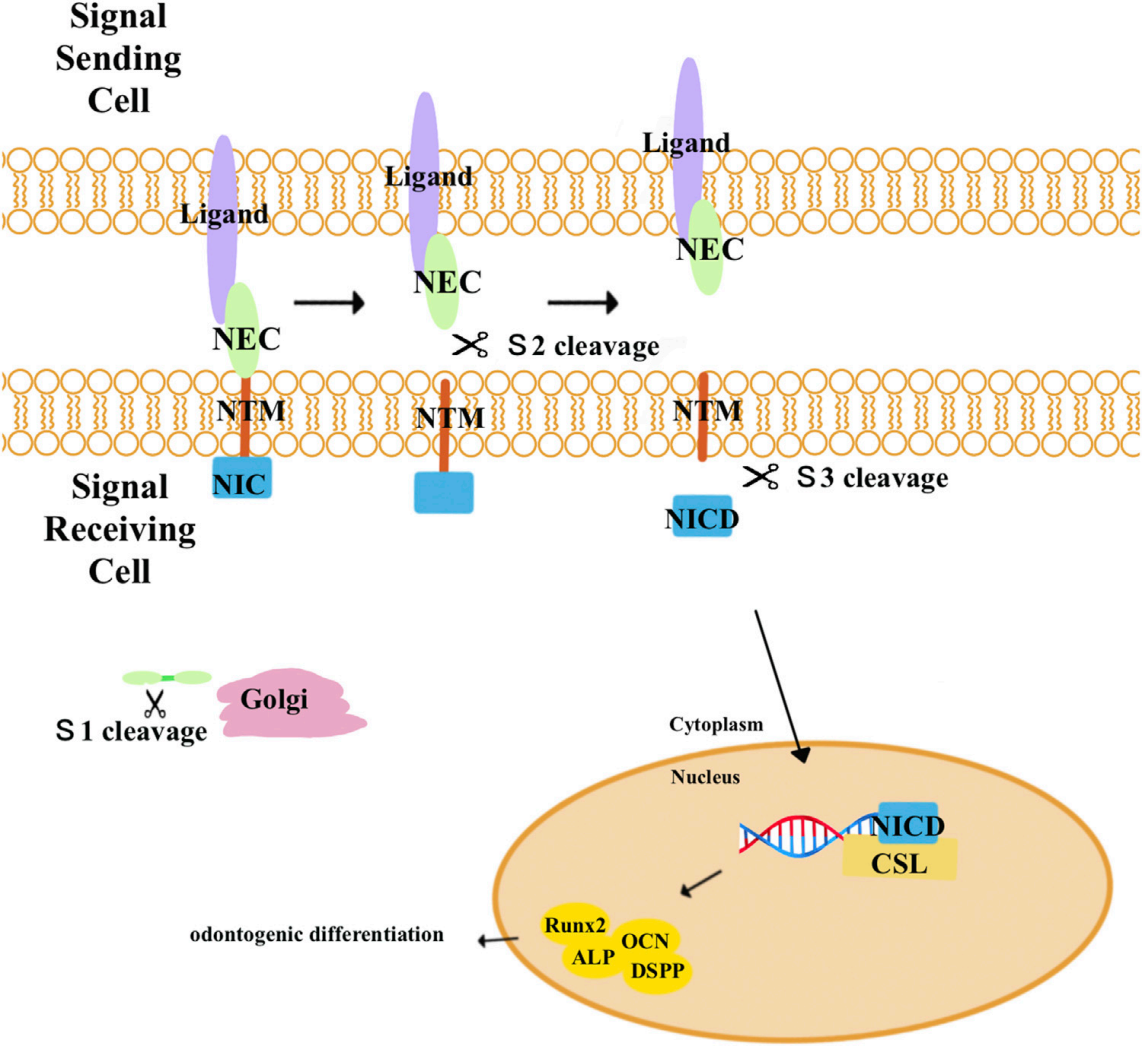
Notch receptors are activated by combination with a ligand from a neighboring cell and undergo three proteolytic cleavages. First, Notch precursor proteins are cleaved (*S1*) by a furin-like convertase in the trans-Golgi apparatus to generate the mature Notch receptor. Composed of Notch extracellular (*NEC*) and Notch transmembrane (*N™*) and an intracellular portion of Notch (*NIC*) subunits, the mature Notch receptor is delivered to the plasma membrane. When the receptors bind to a ligand from a neighboring cell, a second cleavage (*S2*) by *ADAM* metalloproteases dissociates the *NEC* from *N™* and *NIC*. Lastly, *N*™ and *NIC* undergo another cleavage by the γ-secretase complex (S3), leading to the release of *NIC*. *NIC* then translocates into the nucleus where it combines with the CBF-1-Suppressor of Hairless/Lag1 (*CSL*) to activate target genes and proteins related to odontogenic differentiation, including *Runx2*, *ALP*, *DSPP*, and *OCN*.

Notch signaling pathways (Figure 6) are highly conserved pathways that enable communication between neighboring cells that influence proliferation, differentiation, and apoptotic events during development (Artavanis-Tsakonas et al., 1999). The Notch signaling pathways include four transmembrane Notch receptors (*Notch-1*, *Notch-2*, *Notch-3*, and *Notch-4*) and two families of ligands (*Delta* and *Jagged*). When a receptor binds to a ligand from a neighboring cell, Notch receptors will undergo proteolytic cleavages and release the Notch intracellular domain (*NICD*). The NICD then moves into the nucleus and combines with members of the CSL (CBF-1, Suppressor of Hairless, and *Lag-1*) transcription factor family to activate *Notch* target genes (Lai, 2004; Espinoza et al., 2013; Nandagopal et al., 2018).

Notch signaling has been found to promote osteo/odontogenic differentiation in human bone marrow MSCs, human periodontal ligament stem cells, human adipose stem cells, and stem cells isolated from human exfoliated deciduous teeth (Lough et al., 2016; Liao et al., 2017; Tian et al., 2017; Bagheri et al., 2018). However, the impact of Notch signaling on osteogenic differentiation remains unclear. Zhang et al. (2008) demonstrated that the activation of Notch signaling pathways by *Jagged-1* inhibited the odontogenic differentiation of DPSCs *in vitro* and *in vivo*. Meanwhile, overexpression of the constitutively activated *Notch-ICD* could also inhibit the differentiation of DPSCs. When DPSCs were engineered to express Jagged-1 (DPSC/Jag) using retroviruses, they showed decreased levels of ALP activity and the formation of calcified nodules and dentin sialoprotein (*DSP*). Two months after transplanting cells into nude mice, the results revealed that control cells formed mineralized tissues, but DPSC/Jag cells did not generate any mineralized tissues *in vivo*. According to their results, since Notch signaling inhibited *DSPP* expression in DPSCs, it increases the possibility that Notch signaling could inhibit *Runx2* transcriptional activities. In contrast, Manokawinchoke et al. discovered that after maintaining the DPSCs on indirect immobilized *Jagged1* surfaces in an osteogenic medium, the odonto/osteogenic differentiation of DPSCs was enhanced (Manokawinchoke et al., 2017). Furthermore, the effects of Notch signaling on the differentiation of DPSCs may vary depending on the specific Notch ligands. He et al. discovered that the activation of Notch signaling pathways by *Delta1* enhanced the proliferation and odontogenic differentiation of DPSCs. The cells transduced with *Delta1* formed more calcified nodules in less time and with enhanced *DSPP* expression (He et al., 2009). Wang et al. revealed that inhibition of *Delta1* could promote odontogenic differentiation of DPSCs *in vitro*. Lentivirus-mediated *Delta1*-RNAi stably knocked down the expression of *Delta1* and Notch signaling. Furthermore, the differentiating capability of DPSCs/Delta1-RNAi into odontoblasts is much higher than that of control groups (Wang et al., 2011).

Notch receptors and ligands were expressed in the dental mesenchyme and kept DPSCs in an undifferentiated state to protect the dental pulp from injury (Mitsiadis et al., 1997; Mitsiadis et al., 1998; Harada et al., 1999). According to the aforementioned studies, different ligands had varying effects on the odontogenic differentiation of DPSCs, which may result from different experimental conditions or stimuli. The exact mechanisms of Notch signaling pathways remain unclear; thus, we need more research to figure out.

### 2.7 Multipath parallel signaling pathways

The process of odontogenic differentiation of DPSCs is considerably complex, involving the activation of multiple signaling pathways simultaneously. However, most studies failed to explore the underlying mechanisms between activated signaling pathways. It is crucial to figure out synergistic effects between different signaling pathways. In this section, we introduce several interactions between different signaling pathways. According to our search, most signaling pathways interact with MAPK signaling pathways. It appears that MAPK signaling pathways play an important role in promoting the odontogenic differentiation of DPSCs. In addition, we need further studies to figure out the detailed mechanisms.

#### 2.7.1 MAPK signaling pathways and Wnt signaling pathways

It has been reported that MAPK signaling pathways and Wnt signaling pathways can interact with each other (Li et al., 2014). Li revealed that *Wnt6* can activate the JNK signaling pathways in DPSCs and promote odontogenic differentiation. *Wnt6* could upregulate the phosphorylation of *JNK*. While treated with the JNK pathway inhibitor (SP600125), the activation of JNK activity and the expression of c-Jun mRNA were decreased. This indicates that *Wnt6* activated JNK signaling in DPSCs. Meanwhile, they discovered that *Wnt6* enhanced the level of *DSPP*, *Runx2*, and *DMP-1* mRNA as well as upregulated the activity of *ALP* and the formation of calcium deposits. However, when DPSCs were treated with SP600125, these effects of *Wnt6* on DPSCs were blocked.

#### 2.7.2 MAPK signaling pathways and Smad signaling pathways

MAPK signaling pathways can interact with the Smad signaling pathway through the phosphorylation process in DPSCs. Kong et al. demonstrated that high extracellular Mg^2+^ could enhance the odontogenic differentiation of DPSCs by activating the ERK/BMP2/Smad signaling pathways. KEGG pathway analysis revealed that after odontogenic differentiation with high extracellular Mg^2+^, genes related to the MAPK and TGF-β signaling pathways in DPSCs were differentially expressed. Compared to DPSCs treated with 0 mM Mg^2+^, those treated with high extracellular Mg^2+^ showed increased levels of *Runx2*, *DMP-1*, *DSPP*, phosphorylated *ERK*, *BMP2*, *BMPR1*, and phosphorylated *Smad1/5/9*. Meanwhile, these effects could be reduced by 2-APB (inhibiting Mg^2+^entry) or U0126 (inhibiting ERK signaling)(Kong et al., 2019). Li et al. confirmed that extracellular Ca^2+^ triggered the *BMP2*-mediated *Smad1/5/8* and *Erk1/2* signaling pathways of DPSCs, and these pathways converged on the *Runx2* gene to control the odontogenic differentiation of DPSCs (Li et al., 2015). In addition, *P38MAPK* has been found to mediate the phosphorylation of Smad3 in rat myofibroblasts (Furukawa et al., 2003). Furthermore, Wang et al. (2006); Li et al. (2022) discovered that *P38MAPK* differentially affected the phosphorylation of *Smad2* and *Smad3* during *TGF-β* signaling and affected the odontogenic differentiation of DPSCs, and *ERK1/2* may be involved in the process.

#### 2.7.3 MAPK signaling pathways and Wnt signaling pathways and Smad signaling pathways

Yang et al. suggested that *P38MAPK* signaling pathways can mediate *BMP2* to enhance the levels of *β-catenin* in DPSCs. By treating DPSCs with *rhBMP2*, they observed an increase in the expression of *β-catenin* in both the cytoplasm and nucleus, as well as overall protein expression. Stimulation with *BMP2* would increase the obtained TOPflash values, indicating that *BMP2* could activate canonical Wnt signaling. They found that *BMP2* could activate *P38MAPK* signaling, and when canonical *BMP* signaling pathways were blocked with LDN193189, the levels of p-*P38MAPK* were decreased. When they blocked *P38MAPK* signaling with SB203580, the levels of *β-catenin* were not changed after *BMP2* stimulation, indicating that blockade of the *P38MAPK* pathway could prevent *BMP2*-induced activation of Wnt signaling pathways and differentiation of DPSCs (Yang et al., 2015). Based on these findings, we infer that MAPK signaling pathways, Wnt signaling pathways, and Smad signaling pathways might have a relationship in the differentiation of DPSCs.

#### 2.7.4 MAPK signaling pathways and PI3K/AKT/mTOR signaling pathways

Li J. et al. (2022) indicated that the ERK and JNK pathways and the PI3K/AKT/mTOR pathways were involved in the AREG-induced differentiation of DPSCs. Treatment with AREG could increase the protein expression levels of p-*ERK*, p-*JNK*, and p-*AKT* in DPSCs. These effects were inhibited by the PI3K, ERK, JNK, and P38 pathway inhibitors as well as reduced the expression of the mineralization markers and mineralized nodule formation in DPSCs. This research indicated that MAPK signaling pathways and PI3K/AKT/mTOR signaling pathways are interconnected in the odontogenic differentiation of DPSCs.

#### 2.7.5 MAPK signaling pathways and NF-κB signaling pathways

He et al. (2017) demonstrated that low concentrations of the inflammatory cytokine (*IFN-γ*) inhibited the odonto/osteogenic differentiation of DPSCs through the activation of the NF-κB and MAPK signaling pathways. However, when the *NF-κB* inhibitor or *MAPK* inhibitors were applied, it was observed that the odonto/osteogenic differentiation of DPSCs was enhanced, both *in vivo* and *in vitro*, in comparison to the group treated with *IFN-γ* alone. This study indicated that a certain factor could promote the odontogenic differentiation of DPSCs through NF-κB and MAPK signaling pathways.

#### 2.7.6 Notch signaling pathways and Wnt signaling pathways

Previous studies have reported on the interplay between the Notch and Wnt signaling pathways (Ross and Kadesch, 2001; Foltz et al., 2002; Brack et al., 2008). Kornsuthisopon et al. proved that *Wnt5A* is involved in the *Jagged1*-induced mineralization in DPSCs (Kornsuthisopon et al., 2022). This indicated that there may be interactions between the Notch and Wnt signaling pathways during the process of odontogenic differentiation in DPSCs. Kornsuthisopon et al. revealed that *Jagged1*-mediated Notch activation enhanced the expression of Wnt-related genes, including *Wnt2B* and *Wnt5A*. Treatment with *Wnt5A* enhances mineralization, indicating its potential involvement in the *Notch*-induced osteo/odontogenic differentiation of DPSCs.

## 3 Conclusion

At present, numerous types of stem cells have shown their clinical therapeutic potential (Kuehnle and Goodell, 2002). DPSCs can be easily isolated with versatile differentiation potential, leading to them being the focus in bone and dental tissue engineering. However, DPSCs rapidly lose their ability to proliferate and multipotent differentiation in *in vitro* culture, emphasizing the need for improvement (Horibe et al., 2014).

Many researchers have conducted mineralization experiments *in vitro* using cell culture and have found that certain factors can increase the expression of odontogenic differentiation markers in DPSCs, as well as the phosphorylation or expression of key molecules in related signaling pathways. It indicated that these factors could be used to activate related signaling pathways to mediate the odontogenic differentiation of DPSCs.

In this review, we introduced six signaling pathways with different effects on the odontogenic differentiation of DPSCs. Wnt and Notch signaling pathways have opposite influences on the odontogenic differentiation of DPSCs by distinct stimuli, and the detailed mechanisms need to be figured out. However, due to the complex mechanisms of the signaling pathways, most studies have only assessed the basic mechanisms *in vitro*. We need to explore the odontoblast differentiation and repair capability of DPSCs through *in vivo* studies to understand the further mechanisms. Meanwhile, through mineralization experiments and gene expression analysis, most studies have only verified that a certain factor triggered or inhibited a certain signaling pathway to influence the odontogenic differentiation of DPSCs. As is known to all, each signaling pathway does not exist in isolation but rather has certain interactions between them. Nevertheless, a few literature works summarize all the signaling pathways related to the odontogenic differentiation of DPSCs.

In brief, gaining a comprehensive understanding of the signaling pathways in the odontogenic differentiation of DPSCs will help researchers discover the most effective materials in promoting the regeneration of the pulp–dentin complex. We need further studies to figure out the detailed mechanisms of signaling pathways through *in vivo* and *in vitro* studies. Similarly, researchers should pay attention to the relationships between parallel multipath signaling pathways, when assessing the influence of different factors on the odontogenic differentiation of DPSCs.

## Abbreviations

DPSCs, Dental pulp stem cells MSCs, Mesenchymal stem cells APC, Adenomatous polyposis coli GSK-3β, Glycogen synthase kinase-3β Ck1, Casein kinase 1 LRP5/6, Low-density lipoprotein receptor-related proteins 5 and 6 PCP, Planar cell polarity Runx2, Runt-related transcription factor 2 SATB2, Special AT-rich sequence-binding protein 2 OPN, Osteopontin ALP, Alkaline phosphatase DKK1, Dickkopf-1 DANCR, Differentiation antagonizing non-protein coding RNA DSPP, Dentin sialophosphoprotein DMP-1, Dentin matrix protein 1 OCN, Osteocalcin COL1A1, Collagen-type I-alpha1 TGF-β, Transforming growth factor-β BMP, Bone morphogenetic protein MAPK, Mitogen-activated protein kinase MAPKK or MEK, MAPK kinase MAPKKK or MEKK, MAPK kinase kinase ERK, Extracellular signal-regulated kinase JNKMAPK, c-Jun N-terminal kinase NF-kB, Nuclear factor-kappa B TAK1, TGFβ-activated kinase 1 IKKβ, IκB kinase-β IKK, IκB kinase TNFR, Tumor necrosis factor receptor NIK, NFκB-inducing kinase OVX, Ovariectomy Sham DPSCs, Sham-operated group IL-1β, Interleukin-1β IL-6, Interleukin-6 PI3K/AKT/mTOR, Phosphoinositide-3-kinase/protein kinase B/mammalian target of rapamycin RTK, Receptor tyrosine kinase PDK1, PI3K-dependent kinase 1 GOQDs, Graphene oxide quantum dots SCAP, Stem cells from apical papilla NICD, Notch intracellular domain DSP, Dentin sialoprotein IFN-γ, Inflammatory cytokine.
